# Test–Retest Reliability of the One-Repetition Maximum (1RM) Strength Assessment: a Systematic Review

**DOI:** 10.1186/s40798-020-00260-z

**Published:** 2020-07-17

**Authors:** Jozo Grgic, Bruno Lazinica, Brad J. Schoenfeld, Zeljko Pedisic

**Affiliations:** 1 Institute for Health and Sport (IHES), Victoria University, Melbourne, Australia; 2 Faculty of Education, Department of Kinesiology, J.J. Strossmayer University, Osijek, Croatia; 3 Department of Health Sciences, Lehman College, Bronx, NY USA

## Abstract

**Background:**

The test–retest reliability of the one-repetition maximum (1RM) test varies across different studies. Given the inconsistent findings, it is unclear what the true reliability of the 1RM test is, and to what extent it is affected by measurement-related factors, such as exercise selection for the test, the number of familiarization trials and resistance training experience.

**Objectives:**

The aim of this paper was to review studies that investigated the reliability of the 1RM test of muscular strength and summarize their findings.

**Methods:**

The PRISMA guidelines were followed for this systematic review. Searches for studies were conducted through eight databases. Studies that investigated test–retest reliability of the 1RM test and presented intra-class correlation coefficient (ICC) and/or coefficient of variation (CV) were included. The COSMIN checklist was used for the assessment of the methodological quality of the included studies.

**Results:**

After reviewing 1024 search records, 32 studies (pooled *n* = 1595) on test–retest reliability of 1RM assessment were found. All the studies were of moderate or excellent methodological quality. Test–retest ICCs ranged from 0.64 to 0.99 (median ICC = 0.97), where 92% of ICCs were ≥ 0.90, and 97% of ICCs were ≥ 0.80. The CVs ranged from 0.5 to 12.1% (median CV = 4.2%). ICCs were generally high (≥ 0.90), and most CVs were low (< 10%) for 1RM tests: (1) among those without and for those with some resistance training experience, (2) conducted with or without familiarization sessions, (3) with single-joint or multi-joint exercises, (4) for upper- and lower-body strength assessment, (5) among females and males, and (6) among young to middle-aged adults and among older adults. Most studies did not find systematic changes in test results between the trials.

**Conclusions:**

Based on the results of this review, it can be concluded that the 1RM test generally has good to excellent test–retest reliability, regardless of resistance training experience, number of familiarization sessions, exercise selection, part of the body assessed (upper vs. lower body), and sex or age of participants. Researchers and practitioners, therefore, can use the 1RM test as a reliable test of muscular strength.

## Key Points

- The 1RM test has good-to-excellent test–retest reliability.
- The reliability of the test seems to be high regardless of resistance training experience, number of familiarization sessions, exercise selection, part of the body assessed (upper vs. lower body), and sex or age of participants.

## Introduction

Muscular strength can be defined as “the ability to exert a force on an external object or resistance” [1]. Higher levels of muscular strength may result in better performance in a range of sport-specific tasks and decrease the risk of injuries in athletes [1]. An adequate level of muscular strength is also needed for a range of activities of daily life. In older adults, for example, greater strength improves physical functioning and quality of life and reduces the risk of falls [2–4]. Higher muscular strength is also associated with a reduced risk of premature mortality [5]. Taking these factors into account, it is not surprising that organizations such as the American College of Sports Medicine (ACSM) and the World Health Organization (WHO) recommend participating in muscular-strengthening activities on a regular basis [6, 7]. Investigating aspects of strength as a muscular quality in relation to performance in different exercise tasks is important from a sports performance perspective. Studying associations of strength with health outcomes, such as mortality risk, chronic disease, and quality of life, is important to advance public health.

Resistance training is the most commonly used exercise intervention for increasing muscular strength [6]. Resistance training can be performed using isometric muscle actions (i.e., with no net change in muscle length), isokinetic muscle actions (i.e., with a constant rate of movement), and, the most commonly selected, dynamic muscle actions (i.e., coupled eccentric and concentric actions) [6]. To determine the efficacy of a given resistance training program, it is paramount to measure the level of strength as accurately as possible. Furthermore, studies that explore the acute effects of resistance exercise on physiological parameters, such as muscle protein synthesis, hormonal responses, muscle soreness, electromyography outcomes, as well as studies on ergogenic effects of supplements, also use muscle strength testing as a basis for their respective exercise protocols [8–13]. Additionally, exercise prescription for repetition ranges in resistance training is also often based on a given percentage of maximal strength values [6], which further highlights the need for an accurate method of testing strength.

In laboratory-based settings, muscular strength is most commonly assessed using isokinetic dynamometers [14]. However, a disadvantage of such tests is the cost of the necessary equipment [14]. Another limitation of isokinetic dynamometers is that they are generally only single-joint-based tests of strength. A commonly used field-based test of strength is the one-repetition maximum (1RM) test [15]. As suggested by the name, the 1RM is defined as the maximal weight that can be lifted once, while maintaining the correct lifting technique [15]. The 1RM test has several distinct advantages over a laboratory-based test. In the 1RM test, eccentric actions are usually coupled with concentric actions, which is more reflective of dynamic muscle actions that are most commonly used in resistance training and of natural movement in most activities of sport and daily living. The 1RM test allows for assessing strength in multi-joint exercises. Given it does not require expensive equipment, it is highly cost-effective. In trained individuals, 1RM test is also commonly performed using the same exercises as in the training sessions, which might reduce the need for prior familiarization with the test. In addition to these advantages over isokinetic dynamometers, the 1RM test has been shown as safe across different populations, even among children, older adults, and clinical individuals [16–18]. Even though 1RM test can be time-consuming when strength is assessed in a large number of participants, many researchers consider it as the “gold standard” test of dynamic strength [15].

Test–retest reliability represents the consistency of results in a given test across repeated measurements [19, 20]. Reliability of strength tests may be influenced by a number of measurement-related factors, as well as by biological and technical variation in performing a given exercise [20]. Low reliability may reduce statistical power and thus increase the probability of a type II error [20]. In the sport and exercise science area, reliability is commonly expressed using the intra-class correlation coefficient (ICC) and the coefficient of variation (CV). A detailed description of ICC and CV as measures of reliability can be found elsewhere [19, 20].

The test–retest reliability of the 1RM test varies significantly across different studies [16, 18, 21–50]. For example, in one study [48], ICC was 0.64, while in another [26], it was 0.99. Similarly, in the Seo et al. [46] study, CV was 0.5%, while in the Ribeiro et al. [40] study, it was 12.1%. Given the inconsistent findings, it is unclear what the true reliability of the 1RM test is and to what extent it is affected by measurement-related factors, such as exercise selection for the test, number of familiarization trials, and resistance training experience. No previous systematic review has summarized evidence on the test–retest reliability of the 1RM dynamic strength assessment. Therefore, this paper aimed to investigate the reliability of the 1RM test reported in individual studies and summarize their findings.

## Methods

### Search Strategy

The Preferred Reporting Items for Systematic Reviews and Meta-Analyses (PRISMA) guidelines were followed for this systematic review [51]. English-language literature searches of PubMed/MEDLINE, Scopus, Academic Search Elite, CINAHL, MasterFILE Premier, PsycINFO, and SPORTDiscus databases were conducted on January 5th 2020 using the following search syntax: (1RM OR “1 RM” OR 1-RM OR “1 repetition maximum” OR “one repetition maximum”) AND (reliability OR repeatability OR reproducibility). To minimize the study selection bias, the searches were performed independently by two authors (JG and BL) of the review.

### Inclusion Criteria

To be included in the review, studies were required to meet the following criteria: (1) published in English and in a peer-reviewed journal, (2) investigated test–retest reliability of the 1RM test, and (3) presented ICC and/or CV values. As suggested by Koo and Li [52], ICC values were deemed to indicate poor (less than 0.50), moderate (0.50 to 0.75), good (0.75 to 0.90), and excellent (> 0.90) reliability. Even though there are no universally accepted thresholds for classifying CV, values lower than 5% are generally deemed acceptable [53].

### Data Extraction

Two authors (JG and BL) of the review independently extracted the following data to an Excel spreadsheet: (1) details regarding the sample (including sample size, age, and resistance training experience), (2) protocol used for the 1RM test (including the warm-up protocol, number of days between the assessments, and rest between attempts), (3) ICC and/or CV values, and (4) any adverse events associated with the 1RM test. Coding files were checked between the authors, and all discrepancies were resolved through discussion and consensus.

### Methodological Quality

To assess the methodological quality of the included studies, Form B of the validated COSMIN checklist was used [54], which is designed for reliability studies. This form has 11 items that refer to reporting of missing items, adequacy of the sample size, number of measurements, measurement administration, time interval between the assessments, similarity of conditions for both measurements, important flaws in the study design, and the reporting of ICCs. Additional details about the form can be found elsewhere [54]. In all of the questions (besides question ten), the answer “yes” corresponds to one point. Question 10 is as follows: “Were there any important flaws in the design or methods of the study?” In this question, the answer “no” corresponds to a point. The maximal score on the checklist is 11. Studies scoring 10 to 11 points were considered as being of “excellent” methodological quality. Studies scoring 7 to 9 points were considered as being of “moderate” quality, while studies that scored less than 7 points were considered as being of “poor” methodological quality. Studies were rated independently by two reviewers (JG and BL). Any observed differences in the assessment between the reviewers were resolved through discussion and mutual agreement. Study quality was not an inclusion/exclusion criterion in this review.

## Results

### Search Results

**Fig. 1:**
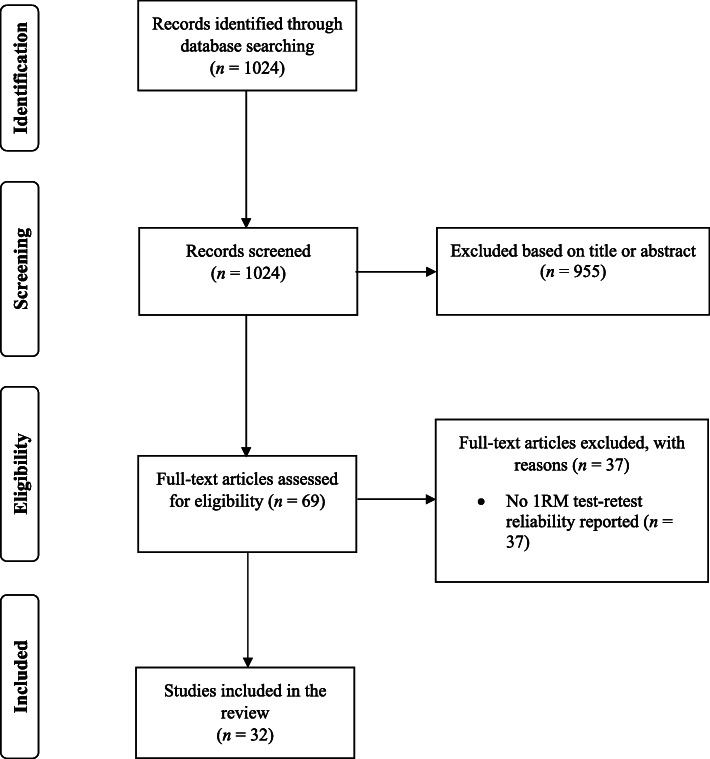
PRISMA flow diagram

The searches through the databases yielded 1024 search results (Fig. 1). Of these, 955 documents were excluded based on their titles and abstracts, while 69 papers were read in full. After assessing the full texts, 37 additional studies were excluded as they did not meet the inclusion criteria. The study selection process, therefore, resulted in the inclusion of 32 studies in this review [16, 18, 21–50].

### Study Characteristics

The pooled number of participants from all included studies was 1595 (median = 35; range = 10–376). Most of the studies were conducted among apparently healthy individuals with two studies examining the reliability of the 1RM test in clinical populations (individuals with Parkinson’s disease and older adults with chronic heart failure [16, 29], respectively). Fourteen studies were conducted among individuals with some resistance training experience, while 22 studies included individuals without any previous resistance training experience (note that four studies included both groups). The period between 1RM test and retest varied between 1 and 10 days. Out of fourteen studies that included familiarization sessions, nine studies used one session, four studies used two sessions, and one study used three familiarization sessions. All but one study presented ICCs, while 15 studies reported CVs (14 studies presented both ICCs and CVs). Table 1 summarizes relevant information pertaining to the included studies.

**Table 1:** Summary of the included studies

Study	Sample	Warm-up protocol	Number of days between the assessments	Rest between attempts	Average or allowed number of 1RM attempts	Number of familiarization sessions	Adverse events
Amarante do Nascimento et al. [21]	45 untrained older women	1 set × 6–10 repetitions (~50% 1RM)	2 days	3–5 min	3 allowed attempts	0	None
Augustsson et al. [22]	30 young resistance-trained men and women	10 min of cycling and 15 squats; 2 sets × 10 repetitions (20 kg); 1 set × 10 repetitions (5 kg)	2–7 days	5 min	Average of 4.5 to 5.7 attempts	1	None
Augustsson et al. [23]	41 young resistance-trained women	5-min cycling; 1 set × 15–20 repetitions (with an empty barbell)	5–9 days	1 min	Median of 4 to 5 attempts	0	None
Barbalho et al. [26]	376 untrained older women	1 set × 8 repetitions (40–50% 1RM); 1 set × 6 repetitions (50–60% 1RM)	2-3 days	5 min	Up to 3 allowed attempts	0	None
Benton et al. [24]	19 young to middle-aged untrained women	2 sets × 5–10 repetitions (~40% 1RM); 1 set × 3–5 repetitions (~60% 1RM)	At least 1 day	2–3 min	Within 3-5 attempts	0	Quadriceps muscle pain in one participant
Benton et al. [25]	10 untrained middle-aged women	1 set × 10 repetitions (~40% 1RM); 1 set × 5 repetitions (~60% 1RM)	At least 1 day	3 min	Within 5 attempts	0	None
Buckley and Hass [16]	46 individuals with Parkinson’s disease (sex not specified)	1 set × 10 repetitions (“low resistance”), with incremental increases in load	At least 3 days	Not presented	Within 5 attempts	2	None
Carabello et al. [27]	57 untrained older men and women with mobility limitations	Not presented	7 days	2 min	Not reported	0	None
Comfort and McMahon [28]	45 male and female collegiate athletes	The National Strength and Conditioning Association protocol	3–5 days	3 min	Up to 6 allowed attempts	0	None
Ellis et al. [29]	24 older adults with chronic heart failure	1 set × 5–10 repetitions (~50% 1RM)	2–5 days	3–5 min	Up to 4 allowed attempts	0	Slight chest discomfort in one participant
Faigenbaum et al. [30]	36 young male athletes	10 min of dynamic movement activities, 5 sets × 1–2 repetitions (~50–90% 1RM)	3–7 days	3 min	Within 3-5 attempts	0	None
García-Ramos et al. [31]	30 young resistance trained men	10 min of jogging, stretching, and shoulder mobilization, 1 set × 5 repetitions (17 kg), and progressive increases in load	At least 2 days	5 min	Not reported	2	None
Grosicki et al. [32]	32 untrained and trained older men and women, and 16 young untrained and trained men and women	1 set with minimal weight, 1 set × 3 repetitions (~40–60% 1RM), 1 set × 3 repetitions (~60–80% 1RM)	At least 2 days	3–5 min	Not reported	1	None
Hageman et al. [33]	31 middle-aged and older untrained women	1 set with minimal weight, 1 set × 3 repetitions (~40–60% 1RM), 1 set × 3 repetitions (~60–80% 1RM)	7 days	3 min	Up to 5 allowed attempts	0	None
LeBrasseur et al. [34]	30 young untrained men, 31 older untrained men, and 39 untrained older men with mobility limitations	Not presented	2–7 days	Not presented	Not reported	0	None
Levinger et al. [18]	53 untrained middle-aged men and women	1 set × 10 repetitions (“light load”), and progressive increases in load	4–8 days	1 min	Within 3–6 attempts	1	Mild soreness in some participants
McCurdy et al. [35]	30 young untrained men and women, and 22 resistance-trained young men and women	1 set × 5–10 repetitions (“light load”), 1 set × 5 repetitions (load increased by 10–20%)	At least 2 days	3–5 min	Within 5 attempts	1	None
McCurdy et al. [36]	16 young resistance-trained men and women	Upper-body stretches; 2 sets × 5 repetitions (“light load”); 1 set × 2–3 repetitions (with increased load)	4 days	2–3 min	Not reported	2	None
Neto et al. [37]	16 young resistance-trained men	3-min cycling and progressive increases in load each warm-up set	3–5 days	5 min	Not reported	0	None
Patterson et al. [38]	58 young resistance-trained men and women	1 set × 6 repetitions (minimal weight on the machine)	3–7 days	1 min	Up to 6 allowed attempts	0	3 participants could not perform the leg press due to lower-body injuries (not clear if the injuries occurred during testing)
Phillips et al. [39]	47 untrained older men and women	5-min cycling; 1 set × 5–10 repetitions (“light load”)	Nonconsecutive days	1–2 min	Not reported	3	None
Ribeiro et al. [40]	24 young untrained men and women	1 set × 6–10 repetitions (~50% 1RM)	2–3 days	3–5 min	Up to 4 allowed attempts	1	None
Ribeiro et al. [41]	67 resistance-trained men, classified as novice, intermediate, or advanced trainees	1 set × 6–10 repetitions (~50% 1RM)	2–3 days	3–5 min	Up to 3 allowed attempts	1	None
Rydwik et al. [42]	23 untrained older men and women, and 11 trained older men and women	5-min walking; 1 set × 10 repetitions (lowest load on the machine)	7 days	1–2 min	Average of 6 attempts	0	Mild soreness in 3 participants
Salem et al. [43]	30 untrained middle aged and older women and men	1 set × 3 repetitions (submaximal load)	7 days	Not reported	Within 3 attempts	0	13 participants reported soreness and one participant aggravated a previous back injury
Schroeder et al. [44]	116 untrained older men	5-min walking or cycling; 1 set × 5 repetitions (~50% 1RM); 1 set × 3–5 repetitions (~75% 1RM)	7–10 days	90 s	Up to 8 allowed attempts	0	Sore joints in 11 participants and fatigue in 2 participants
Scott et al. [45]	13 resistance-trained men	5-min cycling; 1 set × 10 repetitions (~50% 1RM); 1 set × 5 repetitions (~70% 1RM); 1 set × 1 repetition (~90% 1RM)	7 days	3 min	Within 3-6 attempts	1	None
Seo et al. [46]	30 young trained men and women	5-min cycling; 1 set × 8–10 repetitions (~50% 1RM)	2 days	1 min	Within 5 attempts	1	None
Sugiura et al. [47]	20 young untrained men	10-min cycling; 2–3 sets with the smallest load	1–3 days	3 min	Not reported	0	None
Tagesson and Kvist [48]	37 untrained young men and women	10-min cycling; 2–3 sets with the smallest load	1–3 days	3 min	Up to 8 allowed attempts	0	Stiffness in hip and knee in two participants and one participant aggravated a previous back injury
Tiggemann et al. [49]	10 untrained young men, 10 recreationally active men, and 10 resistance-trained men	1 set × 12 repetitions	2–4 days	3–5 min	Within 5 attempts	2	None
Urquhart et al. [50]	14 untrained young men	2 sets × 10 squats, 10 lunges, and 10 butt kicks; 1 set × 5 repetitions (60% 1RM); 1 set × 3 repetitions (75% 1RM); 1 set × 2 repetitions (85% 1RM); 1 set × 1 repetition (90% 1RM); 1 set × 1 repetition (100% 1RM)	4–5 days	4 min	Up to 4 allowed attempts	1	None

*1RM* one-repetition maximum

### 1RM Test Protocols

Out of the studies that detailed their respective warm-up protocols, 16 studies used one submaximal set, 10 studies used two or three submaximal sets, and 2 studies used five submaximal sets for the warm-up (Table 1). Submaximal sets were most commonly performed with loads ranging from 40 to 80% of estimated 1RM. The repetition range in the submaximal sets generally ranged from 1 to 10 repetitions. Eleven studies also incorporated some form of light aerobic exercise during the warm-up (e.g., 5 min of cycling; Table 1). The number of 1RM attempts per testing session ranged from 3 to 8, with 1 to 5 min of rest between attempts.

### Methodological Quality

Based on the COSMIN checklist, all studies were classified as either having excellent (17 studies) or moderate (15 studies) methodological quality. The mean ± standard deviation values of the checklist were 9 ± 1 points (range = 8 to 11 points). The results of the quality assessment can be found in Table 2.

**Table 2:** Results of the methodological quality assessment using the COSMIN checklist

Study	Item 1	Item 2	Item 3	Item 4	Item 5	Item 6	Item 7	Item 8	Item 9	Item 10	Item 11	Total score
Amarante do Nascimento et al. [21]	Yes	Yes	Yes	Yes	No	Yes	Yes	Yes	Yes	No	Yes	10
Augustsson et al. [22]	Yes	Yes	No	Yes	Unclear	Yes	Yes	Yes	Yes	No	Yes	9
Augustsson et al. [23]	Yes	Yes	No	Yes	Yes	Yes	Yes	Yes	Yes	No	Yes	10
Barbalho et al. [26]	Yes	Yes	Yes	Yes	Yes	Yes	Yes	Yes	Yes	No	Yes	11
Benton et al. [24]	Yes	Yes	No	Yes	No	Yes	Yes	Yes	Yes	No	Yes	9
Benton et al. [25]	Yes	Yes	No	Yes	No	Yes	Yes	Yes	Yes	No	Yes	9
Buckley and Hass [16]	Yes	Yes	Yes	Yes	Unclear	Yes	Yes	Yes	Yes	No	Yes	10
Carabello et al. [27]	Yes	Yes	Yes	Yes	Unclear	Yes	Yes	Yes	Yes	No	Yes	10
Comfort and McMahon [28]	Yes	Yes	Yes	Yes	Unclear	Yes	Yes	Yes	Yes	No	Yes	10
Ellis et al. [29]	Yes	Yes	No	Yes	Unclear	Yes	Yes	Yes	Yes	No	Yes	9
Faigenbaum et al. [30]	Yes	Yes	Yes	Yes	Yes	Yes	Yes	Yes	Yes	No	Yes	11
García-Ramos et al. [31]	Yes	Yes	No	Yes	Unclear	Yes	Yes	Yes	Yes	No	Yes	9
Grosicki et al. [32]	Yes	Yes	Yes	Yes	Unclear	Yes	Yes	Yes	Yes	No	Yes	10
Hageman et al. [33]	Yes	Yes	No	Yes	Yes	Yes	Yes	Yes	Yes	No	Yes	10
LeBrasseur et al. [34]	Yes	Yes	Yes	Yes	Unclear	Yes	Yes	Yes	Yes	No	Yes	10
Levinger et al. [18]	Yes	Yes	Yes	Yes	Unclear	Yes	Yes	Yes	Yes	No	Yes	10
McCurdy et al. [35]	Yes	Yes	Yes	Yes	Unclear	Yes	Yes	Yes	Yes	No	Yes	10
McCurdy et al. [36]	Yes	Yes	No	Yes	Unclear	Yes	Yes	Yes	Yes	No	Yes	9
Neto et al. [37]	Yes	Yes	No	Yes	Unclear	Yes	Yes	Yes	Yes	No	Yes	9
Patterson et al. [38]	Yes	Yes	Yes	Yes	Unclear	Yes	Yes	Yes	Yes	No	Yes	10
Phillips et al. [39]	Yes	Yes	Yes	Yes	Unclear	Yes	Yes	Yes	Yes	No	No	9
Ribeiro et al. [40]	Yes	Yes	No	Yes	Unclear	Yes	Yes	Yes	Yes	No	Yes	9
Ribeiro et al. [41]	Yes	Yes	Yes	Yes	Unclear	Yes	Yes	Yes	Yes	No	Yes	10
Rydwik et al. [42]	Yes	Yes	Yes	Yes	No	Yes	Yes	Yes	Yes	No	Yes	10
Salem et al. [43]	Yes	Yes	Yes	Yes	Unclear	Yes	Yes	Yes	Yes	No	Yes	10
Schroeder et al. [44]	Yes	Yes	No	Yes	Unclear	Yes	Yes	Yes	Yes	No	Yes	9
Scott et al. [45]	Yes	Yes	No	Yes	Yes	Yes	Yes	Yes	Yes	No	Yes	10
Seo et al. [46]	Yes	Yes	No	Yes	Unclear	Yes	Yes	Yes	Yes	No	Yes	9
Sugiura et al. [47]	Yes	Yes	No	Yes	Unclear	Yes	Yes	Yes	Yes	No	Yes	9
Tagesson and Kvist [48]	Yes	Yes	No	Yes	Unclear	Yes	Yes	Yes	Yes	No	Yes	9
Tiggemann et al. [49]	Yes	Yes	No	Yes	Unclear	Yes	Yes	Yes	Yes	No	Yes	9
Urquhart et al. [50]	Yes	Yes	No	Yes	Unclear	Yes	Yes	Yes	Yes	No	Yes	9

### Overall Reliability of 1RM Test

**Fig. 2:**
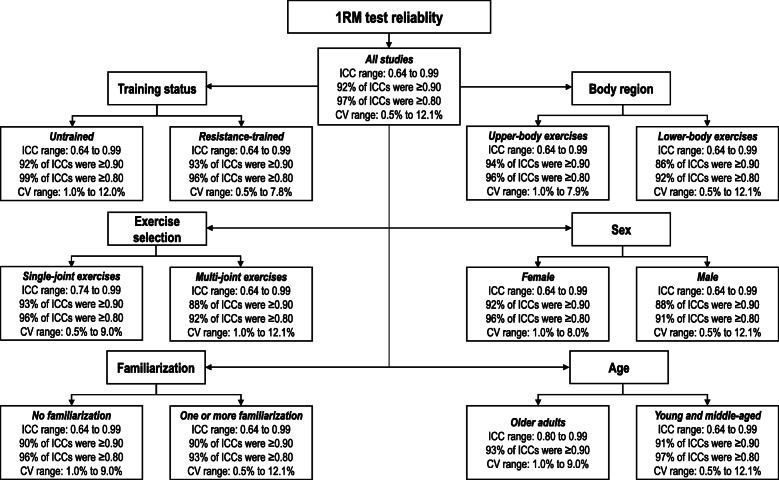
Summary of test–retest reliability of 1RM assessment

Test–retest reliability of 1RM assessment is summarized in Table 3 and Fig. 2. When considering all available studies, ICCs ranged from 0.64 to 0.99 (median ICC = 0.97), where 92% of ICCs were ≥ 0.90, and 97% of ICCs were ≥ 0.80. The range of reported CVs was from 0.5 to 12.1% (median CV = 4.2%).

**Table 3:** Summary of reliability data from the included studies

Study	ICC	ICC type	CV	Systematic changes
Amarante do Nascimento et al. [21]	Chest press: 0.95–97 Biceps curl machine: 0.95–97 Knee extension: 0.95–97	Unclear	Not presented	Chest press: 0.6 kg Biceps curl machine: 0.5 kg Knee extension: 1.2 kg
Augustsson et al. [22]	Knee extension (men): 0.93 Knee extension (women): 0.93	2,1	Knee extension (men): 7.8% Knee extension (women): 6.4%	↔ in any exercise
Augustsson et al. [23]	*Inter-rater* Bench press: 0.98 (95% CI: 0.94–0.99) Back squat: 0.85 (95% CI: 0.40–0.95)	2,1	Not presented	Bench press: ↔ Back squat: 6.8 kg
Barbalho et al. [26]^a^	*Before 12 weeks of training* Bench press: 0.99 Leg press: 0.99 *After 12 weeks of training* Bench press: 0.99 Leg press: 0.99	Unclear	*Before 12 weeks of training* Bench press: < 1% Leg press: < 1% *After 12 weeks of training* Bench press: < 1% Leg press: < 1%	n/a
Benton et al. [24]	Chest press: 0.95 (95% CI: 0.90–0.98) Leg press: 0.95 (95% CI: 0.89–0.98)	Unclear	Not presented	Chest press: ↔ Leg press: 6.9 ± 0.6 kg
Benton et al. [25]^a^	*Before 8 weeks of training* Chest press: 0.98 Leg press: 0.99 *After 8 weeks of training* Chest press: 0.97 Leg press: 0.99	Unclear	Not presented	↔ in any exercise
Buckley and Hass [16]	Chest press: 0.95 (95% CI: 0.90–0.98) Biceps curl machine: 0.97 (95% CI: 0.92–0.98) Knee extension: 0.96 (95% CI: 0.93–0.97) Knee flexion: 0.91 (95% CI: 0.79–0.96)	“2-way random-effects”	Not presented	Chest press: ↔ Biceps curl machine: 2.7 kg (95% CI: 1.2–4.1 kg) Knee extension: 4.0 kg (95% CI: 1.9–6.2 kg) Knee flexion: 2.4 kg (95% CI: 0.2–4.7 kg)
Carabello et al. [27]	Knee extension: 0.80	“2-way fixed-model”	Not presented	n/a
Comfort and McMahon [28]	Back squat (men): 0.99 Back squat (women): 0.97 Power clean (men): 0.99 Power clean (women): 0.99	“2-way random-effects”	Not presented	↔ in any exercise
Ellis et al. [29]	*Intra-rater* Leg press: 0.96 (95% CI: 0.81–1.00) *Inter-rater* Leg press: 0.93 (95% CI: 0.83–0.97)	2,1	Not presented	n/a
Faigenbaum et al. [30]	Power clean: 0.98 (95% CI: 0.96–0.99)	2,*k*	Not presented	Power clean: ↔
García-Ramos et al. [31]	Bench press: 0.98 (95% CI: 0.96–0.99)	Unclear	Bench press: 1.9% (95% CI: 1.5–2.5%)	Bench press: ↔
Grosicki et al. [32]	Biceps curl (older women): 0.90 Biceps curl (young women): 0.93 Biceps curl (older men): 0.96 Biceps curl (young men): 0.98 Leg press (older women): 0.99 Leg press (young women): 0.97 Leg press (older men): 0.98 Leg press (young men): 0.99 Knee extension (older women): 0.91 Knee extension (young women): 0.97 Knee extension (older men): 0.98 Knee extension (young men): 0.94	“1-way random model”	Not presented	Biceps curl (older women): 1.1 kg Biceps curl (young women): 1.4 kg Biceps curl (older men): 1.0 kg Biceps curl (young men): ↔ Leg press (older women): 6.1 kg Leg press (young women): 17.5 kg Leg press (older men): 9.3 kg Leg press (young men): 10.8 kg Knee extension (older women): 2.0 kg Knee extension (young women): 2.5 kg Knee extension (older men): 2.3 kg Knee extension (young men): 8.5 kg
Hageman et al. [33]	Bench press: 0.94 Knee extension: 0.91	3,1	Not presented	n/a
LeBrasseur et al. [34]	Chest press (young men): 0.99 (95% CI: 0.99–1.00) Chest press (older men): 0.98 (95% CI: 0.96–0.99) Chest press (older men with mobility limitations): 0.98 (95% CI: 0.96–0.99) Leg press (young men): 0.98 (95% CI: 0.97–0.99) Leg press (older men): 0.95 (95% CI: 0.88–0.98) Leg press (older men with mobility limitations): 0.98 (95% CI: 0.96–0.99)	“2-way mixed-model for repeated measures”	Not presented	↔ in any exercise
Levinger et al. [18]	Chest press: 0.99 Lat pull down: 0.99 Triceps extension: 0.98 Biceps curl: 0.98 Seated row: 0.99 Leg press: 0.99 Knee extension: 0.97	2,1	Chest press: 6.5% (95% CI: 5.4–8.1%) Lat pull down: 3.4% (95% CI: 2.8–4.2%) Triceps extension: 5.3% (95% CI: 4.4–6.6%) Biceps curl: 7.2% (95% CI: 6.0–7.6%) Seated row: 3.4% (95% CI: 2.8–4.2%) Leg press: 3.3% (95% CI: 2.8–4.1%) Knee extension: 6.0 (95% CI: 6.0–9.0)	Chest press: ↔ Lat pull down: ↔ Triceps extension: ↔ Biceps curl: ↔ Seated row: ↔ Leg press: 3.3 kg Knee extension: ↔
McCurdy et al. [35]	Unilateral squat (untrained men): 0.99 Unilateral squat (untrained women): 0.97 Unilateral squat (trained men): 0.98 Unilateral squat (trained women): 0.99	Unclear	Not presented	Unilateral squat (untrained men): 2.8 ± 3.2 kg Unilateral squat (untrained women): 1.8 ± 2.6 kg Unilateral squat (trained men): 7.1 ± 3.8 kg Unilateral squat (trained women): 1.1 ± 1.5 kg
McCurdy et al. [36]	Chain loaded bench press (men): 0.99 Chain loaded bench press (women): 0.93	Unclear	Chain loaded bench press (men): 1.4% Chain loaded bench press (women): 3.5%	Chain loaded bench press (men): 2.6 kg Chain loaded bench press (women): ↔
Neto et al. [37]	Bench press: 0.97 Lat pull down: 0.93 Triceps extension: 0.96 Biceps curl: 0.94 Knee extension: 0.92 Knee flexion: 0.99 Leg press: 0.96 Half-squat: 0.91	“2-way random model, consistency option, single measures”	Not presented	↔ in any exercise
Patterson et al. [38]	Chest press (men): 0.96 Chest press (women): 0.98 Lat pull down (men): 0.92 Lat pull down (women): 0.98 Shoulder press (men): 0.98 Shoulder press (women): 0.97 Knee extension (men): 0.74 Knee extension (women): 0.97 Leg press (men): 0.69 Leg press (women): 0.91	Unclear	Not presented	↔ in any exercise
Phillips et al. [39]	Not presented	n/a	Bench press (men): 5.4% (95% CI: 4.0–8.7%) Bench press (women): 5.2% (95% CI: 4.3–7.3%) Leg press (men): 6.7% (95% CI: 5.1–10.9%) Leg press (women): 6.3% (95% CI: 5.1–10.9%)	Bench press (men): 4.9 kg Bench press (women): ↔ Leg press (men): ↔ Leg press (women): ↔
Ribeiro et al. [40]	Bench press (men): 0.96 Bench press (women): 0.97 Biceps curl (men): 0.98 Biceps curl (women): 0.98 Smith machine squat (men): 0.77 Smith machine squat (women): 0.89	Unclear	Bench press (men): 6.5% Bench press (women): 5.6% Biceps curl (men): 4.1% Biceps curl (women): 5.3% Smith machine squat (men): 12.1% Smith machine squat (women): 8.0%	Bench press (men): 2.5 kg Bench press (women): ↔ Biceps curl (men): ↔ Biceps curl (women): ↔ Smith machine squat (men): 10 kg Smith machine squat (women): ↔
Ribeiro et al. [41]	Bench press (novice): 0.99 Bench press (intermediate): 0.98 Bench press (advanced): 0.97 Biceps curl (novice): 0.97 Biceps curl (intermediate): 0.98 Biceps curl (advanced): 0.96 Smith machine squat (novice): 0.95 Smith machine squat (intermediate): 0.93 Smith machine squat (advanced): 0.95	Unclear	Bench press (novice): 2.7% Bench press (intermediate): 4.1% Bench press (advanced): 3.4% Biceps curl (novice): 3.8% Biceps curl (intermediate): 3.8% Biceps curl (advanced): 3.5% Smith machine squat (novice): 5.4% Smith machine squat (intermediate): 6.5% Smith machine squat (advanced): 6.3%	Bench press (novice): 2.2 kg Bench press (intermediate): 3.5 kg Bench press (advanced): 3.2 kg Biceps curl (novice): 1.8 kg Biceps curl (intermediate): 2.1 kg Biceps curl (advanced): 2.0 kg Smith machine squat (novice): 8.6 kg Smith machine squat (intermediate): 10.9 kg Smith machine squat (advanced): 9.9 kg
Rydwik et al. [42]	Shoulder press (untrained): 0.97 Shoulder press (trained): 0.97	Unclear	Not presented	Shoulder press (untrained): 1.3 kg (95% CI: 0.1–2.5 kg) Shoulder press (trained): 1.7 kg (95% CI: 0.5–2.4 kg)
Salem et al. [43]	Leg press: 0.98 Knee extension: 0.98 Knee flexion: 0.96 Ankle plantar flexion: 0.97	Cronbach’s alpha	Ankle plantar flexion: 6.0%	Leg press: 9.1 kg Knee extension: ↔ Knee flexion: 2.7 kg Ankle plantar flexion: 3 kg
Schroeder et al. [44]^b^	Chest press (cohort 1): 0.94 Chest press (cohort 2): 0.91 Lat pull down (cohort 1): 0.91 Lat pull down (cohort 2): 0.92 Leg press (cohort 1): 0.90 Leg press (cohort 2): 0.96 Knee extension (cohort 1): 0.88 Knee extension (cohort 2): 0.98 Knee flexion (cohort 1): 0.84 Knee flexion (cohort 2): 0.93	Unclear	Chest press (cohort 1): 5.3% Chest press (cohort 2): 7.9% Lat pull down (cohort 1): 5.9% Lat pull down (cohort 2): 4.4% Leg press (cohort 1): 6.3% Leg press (cohort 2): 4.2% Knee extension (cohort 1): 9.0% Knee extension (cohort 2): 4.7% Knee flexion (cohort 1): 7.8% Knee flexion (cohort 2): 7.0%	Chest press (cohort 1): ↔ Chest press (cohort 2): ↔ Lat pull down (cohort 1): ↔ Lat pull down (cohort 2): ↔ Leg press (cohort 1): ↔ Leg press (cohort 2): ↔ Knee extension (cohort 1): 3 ± 7 Knee extension (cohort 2): 3 ± 4 Knee flexion (cohort 1): ↔ Knee flexion (cohort 2): ↔
Scott et al. [45]	Harness back squat: 0.98 (95% CI: 0.93, 0.99)	Unclear	Harness back squat: 2.6% (95% CI: 1.9–4.3%)	Harness back squat: ↔
Seo et al. [46]	Bench press (men): 1.00 Bench press (women): 1.00 Lat pull down (men): 0.98 Lat pull down (women): 1.00 Shoulder press (men): 0.64 Shoulder press (women): 1.00 Triceps extension (men): 1.00 Triceps extension (women): 1.00 Seated row (men): 0.84 Seated row (women): 1.00 Biceps curl (men): 0.99 Biceps curl (women): 1.00 Leg press (men): 1.00 Leg press (women): 1.00 Squat (men): 0.99 Squat (women): 0.97 Knee extension (men): 1.00 Knee extension (women): 0.99 Knee flexion (men): 1.00 Knee flexion (women): 0.99 Hip flexion (men): 0.97 Hip flexion (women): 1.00 Hip extension (men): 1.00 Hip extension (women): 1.00	Unclear	Bench press (men): 2.9% Bench press (women): 5.4% Lat pull down (men): 2.1% Lat pull down (women): 4.4% Shoulder press (men): 2.2% Shoulder press (women): 4.4% Triceps extension (men): 3.0% Triceps extension (women): 5.5% Seated row (men): 2.3% Seated row (women): 4.8% Biceps curl (men): 3.3% Biceps curl (women): 6.4% Leg press (men): 2.4% Leg press (women): 3.2% Squat (men): 3.5% Squat (women): 5.3% Knee extension (men): 1.6% Knee extension (women): 3.1% Knee flexion (men): 1.2% Knee flexion (women): 3.3% Hip flexion (men): 1.7% Hip flexion (women): 3.1% Hip extension (men): 0.5% Hip extension (women): 2.0%	↔ in any exercise
Sugiura et al. [47]	*Intra-rater* Knee extension: 0.99 *Inter-rater* Knee extension: 0.98	1,1 and 2,1	Not presented	n/a
Tagesson and Kvist [48]	*Intra-rater* Squat: 0.64 Knee extension: 0.90 *Inter-rater* Squat: 0.94 Knee extension: 0.96	2,1	Not presented	*Intra-rater* Squat: ↔ Knee extension: ↔ *Inter-rater* Squat: 4.2 ± 7.3 kg Knee extension: ↔
Tiggemann et al. [49]	Bench press (untrained men): 0.99 Bench press (recreationally active men): 0.99 Bench press (trained men): 1.00 Leg press (untrained men): 0.93 Leg press (recreationally active men): 0.97 Leg press (trained men): 0.92	Unclear	Not presented	Bench press (untrained men): 1.2 kg Bench press (recreationally active men): 0.6 kg Bench press (trained men): 1.4 kg Leg press (untrained men): 7 kg Leg press (recreationally active men): 5.5 kg Leg press (trained men): 14.9 kg
Urquhart et al. [50]	Single leg squat: 0.99 (0.96–1.00)	Unclear	1.6% (1.1–2.6%)	Single leg squat: 2.1 kg

*ICC* interclass correlation; *CV* coefficient of variation; *1RM* one-repetition maximum; *ICC 1,1* one-way random effects, absolute agreement, single rater/measurement; *ICC 2,1* two-way random effects, absolute agreement, single rater/measurement; *ICC 2,k* two-way random effects, absolute agreement, multiple raters/measurements; *ICC 3,1* two-way mixed effects, consistency, single rater/measurement

^a^Tested at the beginning of a training program and after a training program

^b^Data from two cohorts

↔ No significant difference

### Reliability in Relation to Training Status and Familiarization

Twenty-two studies included untrained individuals. ICCs for 1RM tests among untrained individuals ranged from 0.64 to 0.99 (median ICC = 0.97), where 92% of ICCs were ≥ 0.90, and 99% of ICCs were ≥ 0.80. The range of reported CVs was from 1 to 12.0% (median CV = 5.5%). Fourteen studies included individuals with some previous resistance training experience. ICCs for 1RM tests among individuals with previous resistance training experience ranged from 0.64 to 0.99 (median ICC = 0.98), where 93% of ICCs were ≥ 0.90, and 96% of ICCs were ≥ 0.80. The range of reported CVs was from 0.5 to 7.8% (median CV = 3.3%).

Eighteen studies did not include a familiarization session. ICCs in these studies ranged from 0.64 to 0.99 (median ICC = 0.96), where 90% of ICCs were ≥ 0.90, and 96% of ICCs were ≥ 0.80. The range of reported CVs was from 1.0 to 9.0% (median CV = 5.3%). Fourteen studies included one or more familiarization sessions. In these studies, ICCs ranged from 0.64 to 0.99 (median ICC = 0.98), where 90% of ICCs were ≥ 0.90, and 93% of ICCs were ≥ 0.80. The range of reported CVs was from 0.5 to 12.1% (median CV = 3.8%).

### Reliability in Relation to Exercise Selection and Body Region

Seventeen studies used single-joint exercises. ICCs for 1RM tests using single-joint exercises ranged from 0.74 to 0.99 (median ICC = 0.97), where 93% of ICCs were ≥ 0.90, and 96% of ICCs were ≥ 0.80. The range of reported CVs was from 0.5 to 9.0% (median CV = 4.1%). Twenty-eight studies used multi-joint exercises. ICCs for 1RM tests using multi-joint exercises ranged from 0.64 to 0.99 (median ICC = 0.98), where 88% of ICCs were ≥ 0.90, and 92% of ICCs were ≥ 0.80. The range of reported CVs was from 1.0 to 12.1% (median CV = 4.3%).

Twenty-one studies assessed upper-body strength. ICCs for 1RM tests of upper-body strength ranged from 0.64 to 0.99 (median ICC = 0.98), where 94% of ICCs were ≥ 0.90, and 96% of ICCs were ≥ 0.80. The range of reported CVs was from 1.0 to 7.9% (median CV = 4.1%). Twenty-eight studies assessed lower-body strength. ICCs for 1RM tests of lower-body strength ranged from 0.64 to 0.99 (median ICC = 0.97), where 86% of ICCs were ≥ 0.90, and 92% of ICCs were ≥ 0.80. The range of reported CVs was from 0.5 to 12.1% (median CV = 4.7%).

### Reliability in Relation to Sex and Age of Participants

Fifteen studies included female participants. ICCs for 1RM tests among females ranged from 0.64 to 0.99 (median ICC = 0.98), where 92% of all ICCs were ≥ 0.90, and 96% of ICCs were ≥ 0.80. The range of reported CVs was from 1.0 to 8.0% (median CV = 4.4%). Nineteen studies included male participants. ICCs for 1RM tests among males ranged from 0.64 to 0.99 (median ICC = 0.97), where 88% of all ICCs were ≥ 0.90, and 91% of ICCs were ≥ 0.80. The range of reported CVs was from 0.5 to 12.1% (median CV = 4.0%).

Twelve studies included older adult participants. ICCs for 1RM tests among older adults ranged from 0.80 to 0.99 (median ICC = 0.97), where 93% of all ICCs were ≥ 0.90. The range of reported CVs was from 1.0 to 9.0% (median CV = 5.4%). Twenty-two studies included young to middle-aged adult participants. ICCs for 1RM tests among young and middle-aged adults ranged from 0.64 to 0.99 (median ICC = 0.98), where 91% of all ICCs were ≥ 0.90, and 97% of ICCs were ≥ 0.80. The range of reported CVs was from 0.5 to 12.1% (median CV = 3.5%).

### Systematic Changes in Results Between Repeated Measurements

In 66% of the analyses that assessed potential systematic changes in 1RM test results between the repeated measurements, no significant changes were found. The remaining studies found higher 1RM values in the retest condition. For lower-body exercises, the reported increases in 1RM ranged from 1.1 to 17.5 kg (median = 5.5 kg). For upper-body exercises, the reported increases in 1RM ranged from 0.5 to 4.9 kg (median = 1.8 kg).

## Discussion

### Main Findings of the Review

The main finding of this systematic review is that the 1RM test generally has excellent test–retest reliability, regardless of the previous resistance training experience, sex, and age of the participants; whether or not the testing procedure includes familiarization sessions; whether the exercises are classified as single- or multi-joint movements; and whether the testing is conducted for upper- or lower-body musculature. This finding is based on 32 included studies that showed either excellent or moderate methodological quality.

### Reliability in Relation to Training Status and Familiarization

Research has established that the response to resistance exercise varies between resistance-trained and untrained individuals [55, 56]. For example, studies have reported differential molecular and epigenetic responses between trained and untrained individuals following an acute bout of resistance exercise [55, 56]. Duez et al. [57] also reported larger action potentials and electric activity of motor units in resistance-trained participants, compared with untrained participants. Accordingly, some authors [58] speculated 1RM test reliability may be different between resistance-trained and untrained individuals. However, when we grouped the ICCs and CVs according to training status, the data showed similar reliability for individuals with and without resistance training experience. These results suggest that resistance training experience might not be as important for the 1RM test as previously thought [58]. From a practical perspective, the results suggest that exercise practitioners may consider using the 1RM test as a reliable test of strength even among untrained participants. Furthermore, the 1RM test seems to be generally safe, as the studies reported very few adverse events associated with the measurement. Most commonly, only muscle soreness was reported (Table 1).

In the Ploutz-Snyder and Giamis study [59], the authors reported that untrained individuals needed as much as eight familiarization sessions with the 1RM test to obtain a reliable measurement. Specifically, these authors reported an average increase in the 1RM test by 13 kg from the first to the final testing session (~1.6 kg per session). They employed a protocol in which the 1RM test was conducted every two days over a period of 2 to 3 weeks. The included participants were required to return to testing if their 1RM on one session exceeded their 1RM on the previous session by 1 kg. Such a strict familiarization procedure might be inefficient and could potentially lead to an increase in the dropout rates of participants. Also, such a testing design might even result in an unwanted training effect, as studies show that merely practicing the 1RM test can produce similar strength gains as high-volume resistance training routine [60]. Studies that did not include any familiarization and studies that included at least one familiarization session showed very high and similar ICC values (over 90% of ICCs were ≥ 0.90). These results suggest that familiarization sessions are not necessary for a reliable assessment of 1RM. While the results would suggest that a familiarization session is likely not required for a reliable 1RM assessment, there may be cases when some familiarization with the exercise to needed, e.g. when a practitioner estimates that the participant's skill in a given exercise is not sufficient and that, therefore, performing the test without further familiarization may increase the risk of injury. To avoid the abovementioned potential issues, in such cases, familiarization can be incorporated into the first testing session, as done by Benton and colleagues [24, 25].

### Reliability in Relation to Exercise Selection and Body Region

Besides training experience, variables such as exercise complexity have been suggested to play an impactful role in the reliability of the 1RM test [48]. For example, one study used the squat and knee extension exercises for the 1RM test [48]. For the squat, which is the more complicated exercise to perform, the ICC was 0.64, while for the knee extension exercise, the ICC was 0.90. However, when examining the whole body of literature, the data for single- and multi-joint exercises showed that the reliability of the 1RM test is high regardless of the resistance exercise selection. Indeed, even studies that assessed the 1RM test using very complex exercises, such as the power clean, reported ICCs of 0.98 and 0.99 [28, 30], albeit these findings are specific to young athletes. Similar results, indicating no substantial differences in reliability, were seen in the subgroup analyses for upper- and lower-body exercises.

### Reliability in Relation to Sex and Age of Participants

Even though there are physiological differences between men and women, especially in muscle contractile properties, fiber type proportion, and perfusion [61], we found no clear indication of a difference in 1RM test reliability between sexes. Research has also established physiological differences in voluntary muscle activation by age, with younger adults having higher muscle activation than their older counterparts [62]. However, we found no clear indication that age affects the test–retest reliability of the 1RM test. It should be noted that making direct comparisons between sex and age groups across different studies is challenging, given that exercise selection and other elements of the testing protocol vary. The evidence base would benefit from more studies that include analyses stratified by sex and age groups within a single study. Nevertheless, the currently available evidence suggests that the 1RM test is a reliable test of muscle strength among both sexes and different age groups.

### Systematic Changes in Results Between Repeated Measurements

Most studies did not find systematic changes in results between the repeated measurements. In those that did, the observed changes were generally small. Their size was well below the average increases in strength commonly found in strength training interventions [63–67]. This is important to consider given that the most common application of the 1RM test is for evaluating changes in strength following a given training program.

### Methodological Quality of Included Studies

The included studies were classified as having excellent or moderate methodological quality based on the COSMIN checklist. While 31 studies presented ICC values and thus received a point on item 11, one study presented only CV values (Table 3). Therefore, future studies should consider presenting ICC coupled with the CV values as both can provide valuable information about reliability. Detailed reasoning for presenting both of the reliability coefficients is available in the paper by Atkinson and Nevill [20]. Despite the moderate-to-excellent quality of the included studies, there is one limitation noted that needs to be highlighted. Namely, not all studies presented the type of ICC used in the analysis. There are ten different types of ICCs that provide different estimates of reliability [52]. When calculated from the same data, one study demonstrated that six different types of ICC ranged from 0.51 to 0.87 [68]. This issue is not limited to the studies included herein as recent reviews that focused on the test–retest reliability of the Yo-Yo test and the 30-15 Intermittent Fitness Test (30-15 IFT) also highlighted this as a limitation [69, 70]. Even though not all studies reported the specific type of ICC types they used, 92% of all ICCs were still ≥ 0.90, suggesting that this limitation might not have had a profound impact on the findings of this review. Nevertheless, future studies conducted on this topic should clearly state which ICC was used for the analysis, to allow for better-informed comparisons of results between studies.

### Recommendations for Future Research

Evidence on the reliability of the 1RM test in clinical populations is scarce, as our search revealed only two such studies. Buckley and Hass [16] included 46 individuals with Parkinson’s disease and explored the reliability of 1RM test assessment of four resistance training exercises. The authors reported ICC values ranging from 0.91 to 0.97. Ellis et al. [29] included individuals with chronic heart failure and reported excellent reliability of the 1RM test for the leg press (ICC = 0.97). These findings would suggest that the 1RM test is a highly reliable test of strength even among clinical individuals. However, the evident lack of studies that explored specific clinical populations highlights the need for future research.

The included studies generally focused on test–retest reliability. However, four studies [23, 29, 47, 48] also provided data for inter-rater reliability. The respective ICCs ranged from 0.85 to 0.98, where 83% of all ICCs were higher than 0.90. Although it seems that the inter-rater reliability of the 1RM test is also high, given that the number of studies was relatively small, this topic should be further explored in future research.

The warm-up protocols varied across the included studies. For example, the studies used between one and five sets with submaximal loads for the warm-up (Table 1). Additionally, some studies also incorporated light aerobic exercise into the warm-up (Table 1). The number of 1RM attempts in some studies was limited (usually to a maximum of three to five attempts), whereas others used progressive increases in the load until the participant could no longer perform a successful 1RM attempt (Table 1). Despite the differences in the warm-up and testing protocols, the reliability of the 1RM test was generally high across all studies. However, future studies may consider exploring the influence of different warm-up strategies and testing protocols on the reliability of 1RM test.

### Limitations of the Review

There are some limitations that need to be considered when interpreting the findings of this review. While there are different statistical measures to express test–retest reliability, the current review focused only on ICC and CV as the two most commonly used reliability coefficients in this research area. Twelve included studies additionally used Bland–Altman plots [18, 21, 23, 28–30, 34, 40, 42, 44, 47, 48] and found relatively narrow 95% limits of agreement (LoA). For example, 95% LoA for the bench press, power clean, leg press, and squat were ± 3–5 kg, ± 5–8 kg, ± 8–13 kg, and ± 10–15 kg, respectively [23, 28–30, 40, 44, 48], which further indicates a high reliability of the 1RM test. However, given the small number of studies that used Bland–Altman plots, future research may also consider using this statistic to provide further insights into LoA for other resistance exercises used for the 1RM test.

## Conclusion

Accurate assessment of strength is the foundation upon which optimal resistance training programs for dynamic strength gains can be developed and evaluated. Based on the results of this review, it can be concluded that the 1RM test generally has good-to-excellent test–retest reliability. The reliability of the 1RM test tends to be excellent regardless of resistance training experience, number of familiarization sessions, exercise selection, part of the body assessed (upper vs. lower body), and sex or age of participants. No or only small systematic changes in 1RM are expected between repeated measurements. Researchers and practitioners can, therefore, use the 1RM test as a reliable test for assessing maximal dynamic muscular strength.

## Data Availability

The datasets analyzed during the current study are available from the corresponding author on reasonable request.
